# Supplementary figures and images for: Reduced prevalence of phage defense systems in Pseudomonas aeruginosa strains from cystic fibrosis patients
Source: mBio. 2025 Feb 25;16(4):e03548-24. doi: 10.1128/mbio.03548-24 (PMC11980395; doi:10.1128/mbio.03548-24)

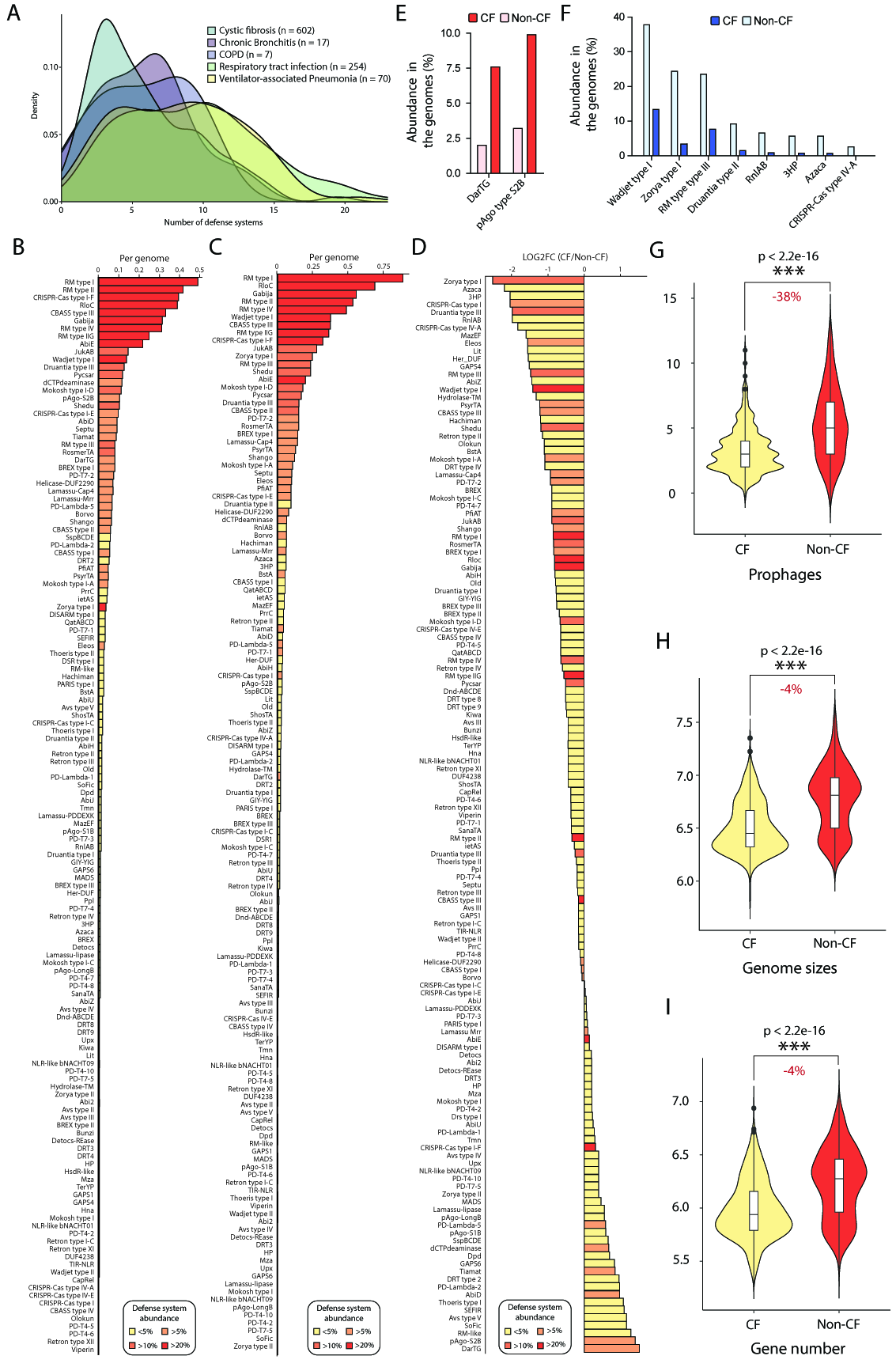

Supplement: Fig. S1 — Comparison of the phage defense system repertoire in Pseudomonas aeruginosa strains isolated from cystic and non-cystic fibrosis lungs. [file mbio.03548-24-s0001.tif]
