# Supplementary material for: Reduced prevalence of phage defense systems in Pseudomonas aeruginosa strains from cystic fibrosis patients
Source: mBio. 2025 Feb 25;16(4):e03548-24. doi: 10.1128/mbio.03548-24 (PMC11980395; doi:10.1128/mbio.03548-24)
Supplement: Supplemental legends — Legends for Figure S1 and Tables S1 and S2. [file mbio.03548-24-s0002.docx]

**Figure S1. Comparison of the phage defense system repertoire in *Pseudomonas aeruginosa* strains isolated from cystic and non-cystic fibrosis lungs.** (A) A density plot of the number of defense systems per *P. aeruginosa* strain isolated from CF lungs and the other non-CF lung sources. (B) The prevalence of individual defense systems per *P. aeruginosa* strain isolated from CF lungs. (C) The prevalence of individual defense systems per *P. aeruginosa* strain isolated from non-CF lungs. (D) The relative log_2_FC change in prevalence of individual defense systems between *P. aeruginosa* strains isolated from CF and non-CF patients. (E) The subset of significantly differentially abundant (adjusted p-value < 0.01) defense systems that are found to be overly abundant (log_2_FC > 1) and (F) less abundant (log_2_FC < -1) in CF isolates. (G) The number of detectable prophages in *P. aeruginosa* strains isolated from CF and non-CF patients. (H) The genome size of *P. aeruginosa* strains isolated from CF and non-CF patients. (I) The number of genes present in *P. aeruginosa* strains isolated from CF and non-CF patients.

**Table S1.** Summary table of the *Pseudomonas aeruginosa* strains and additional information, including the defense system repertoire.

**Table S2.** The relative fold changes in defense system prevalence in *Pseudomonas aeruginosa* strains isolated from CF lungs compared to those from non-CF lungs
